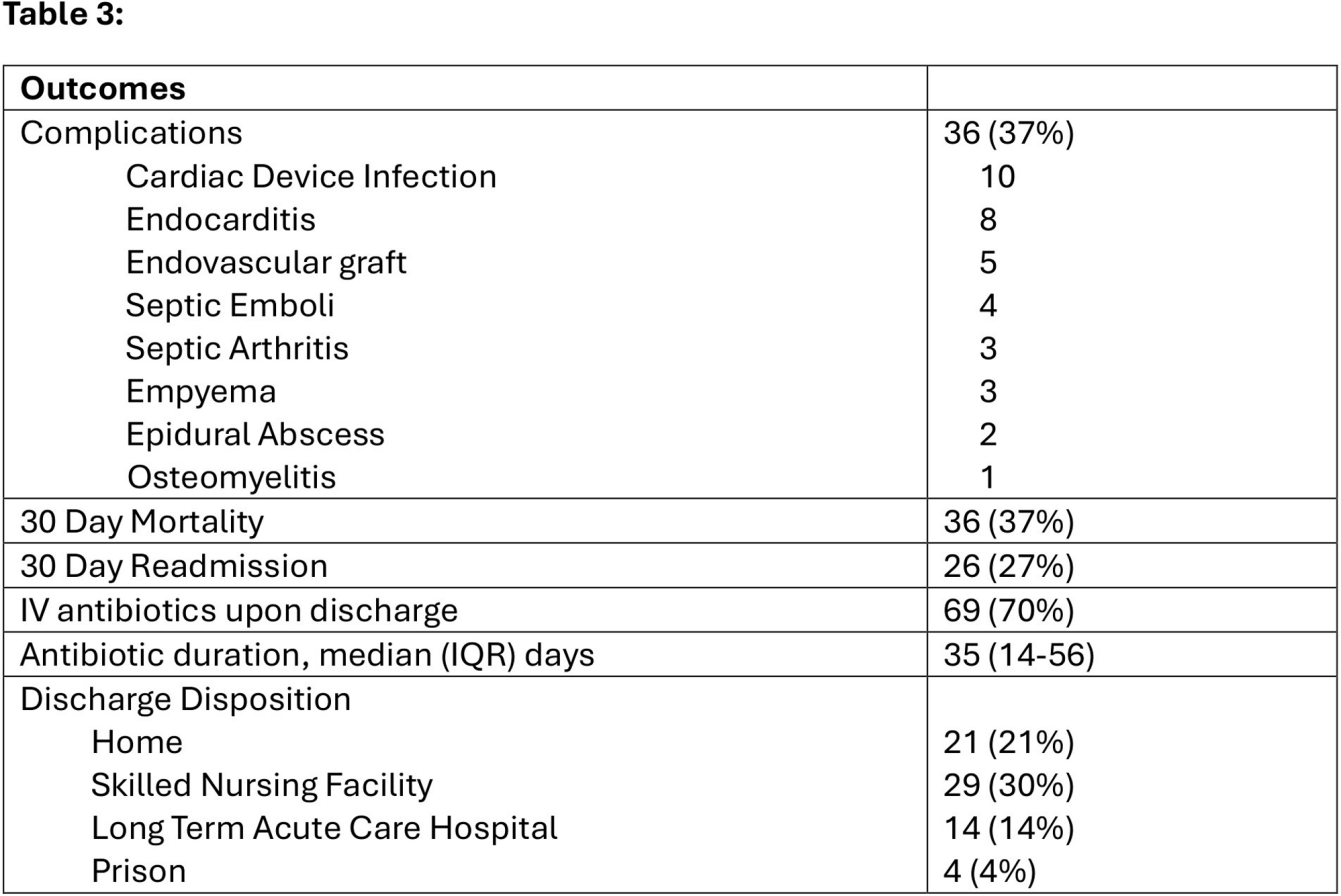# 312 Factors Distinguishing Carbapenemase-producing Carbapenem-resistant Enterobacterales (CP-CRE) from non-CP-CRE

**DOI:** 10.1017/ash.2026.10663

**Published:** 2026-06-23

**Authors:** Douglas Kepko, Nora Colburn, Chelsea Fauver, Shandra Day

**Affiliations:** 1 Ohio State University; 2 The Ohio State University; 3 The Ohio State University Wexner Medical Center

## Abstract

**Background:** Hospital onset bacteremia (HOB) due to Methicillin Resistant Staphylococcus aureus (MRSA), defined as a positive blood culture obtained on admission day 4 or later, is associated with increased patient morbidity and mortality as well as excess healthcare utilization and costs. Determining patient risk factors as well as the most common sources of MRSA HOB can help identify patients at increased risk and guide infection prevention opportunities. **Methods:** A retrospective descriptive cohort study of patients with MRSA HOB was conducted at a quaternary care hospital from July 1, 2023 to June 30, 2025. Demographic and clinical data collected included comorbidities, hospital length of stay (LOS), chlorhexidine gluconate (CHG) treatment, presence of central venous catheter, and MRSA colonization/infection or hospitalization in prior 12 months. Clinical outcome data collected included infectious complications, need for intravenous (IV) antibiotics, IV treatment duration, 30 day mortality, 30 day readmission and discharge location. The primary source of MRSA bacteremia was determined by review of clinical chart documentation. **Results:** During the study period, 98 cases of MRSA HOB were identified. Demographics, clinical data and co-morbidities are detailed in Table 1. Primary sources of the MRSA bacteremia are detailed in Table 2 with the most common being vascular catheters, skin and soft tissue and pneumonia. Patient outcomes including infectious complications, 30 day mortality and 30 day readmission are detailed in Table 3. Overall healthcare worker hand hygiene compliance, measured via electronic hand hygiene monitoring system, was 83.3%. Discussion: Patients with MRSA HOB had several pre-existing co-morbidities with high exposure to the healthcare system. Less than half were found to have prior MRSA colonization/infection, but this is likely an underestimate since our institution does not perform active screening surveillance for MRSA. A majority of patients had a preventable source of bacteremia (vascular catheters and pneumonia) indicating opportunities for improvement with daily CHG treatment, hand hygiene and vascular access maintenance bundles. Proposed strategies to reduce hospital acquired pneumonia include improving oral care compliance and increasing patient mobility although further studies are needed. Patients with MRSA HOB had high rates infectious complications and over half required care in a facility post-discharge. More than a third of patients expired within 30 days of discharge. Targeted prevention strategies for MRSA HOB are needed and have the potential for significant impact in morbidity and mortality.

**Figure:**
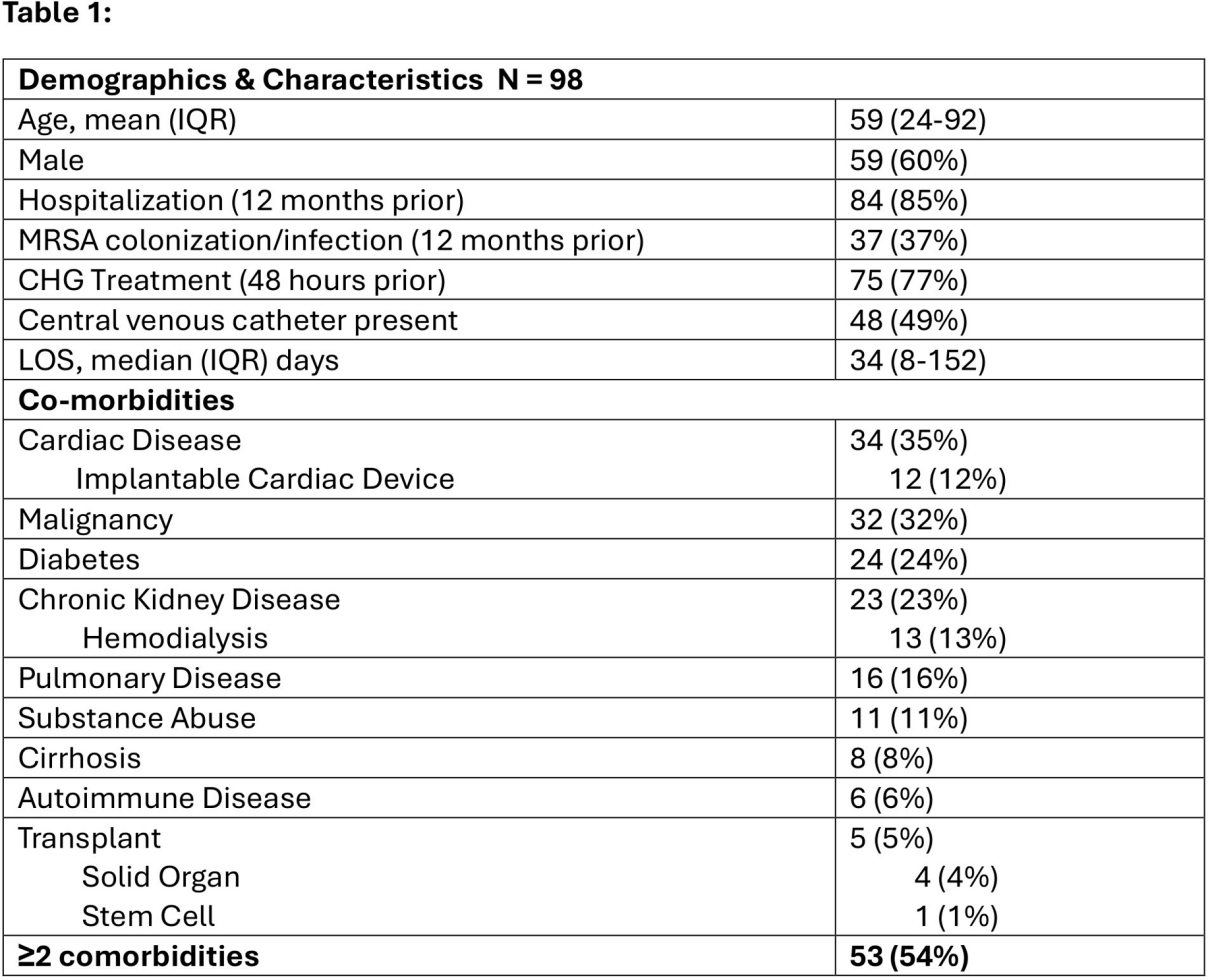

**Figure:**
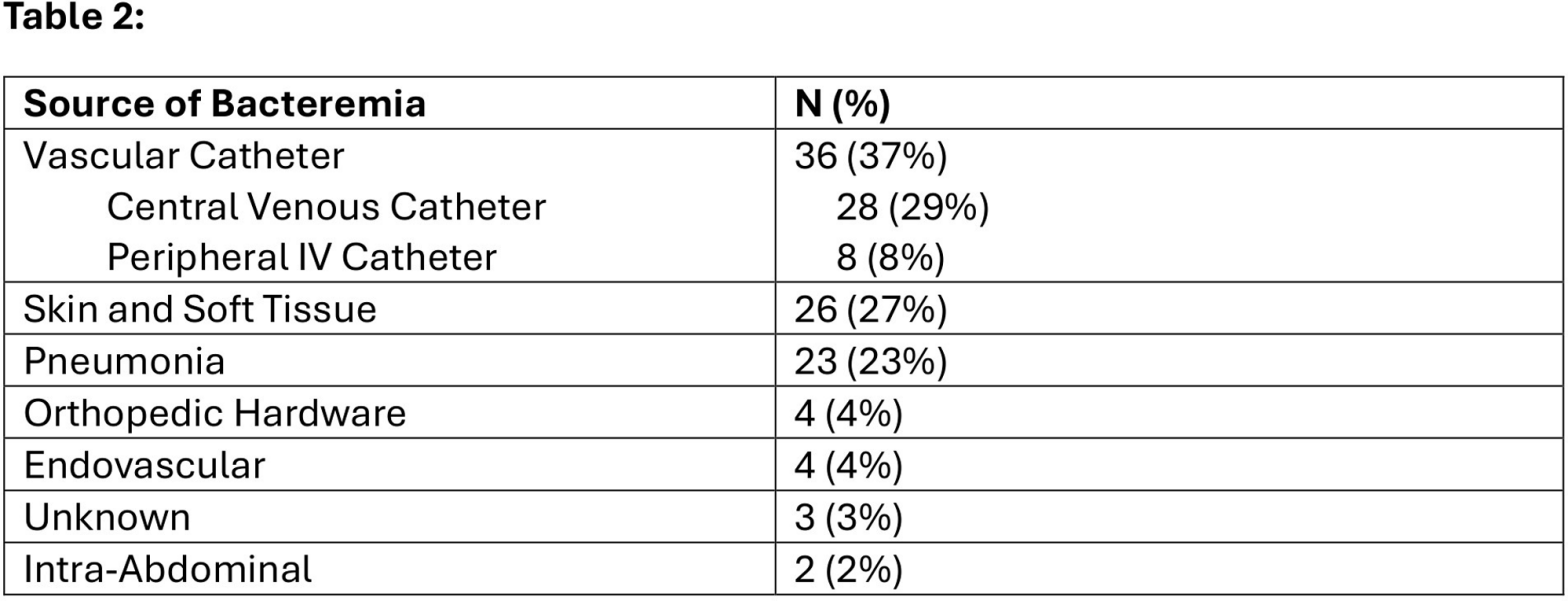

**Figure:**